# Impact of COVID-19 Containment Measures on Unemployment: A Multi-country Analysis Using a Difference-in-Differences Framework

**DOI:** 10.34172/ijhpm.2022.7036

**Published:** 2023-01-31

**Authors:** Walter Morris, Ana Correa, Rolando Leiva

**Affiliations:** Institute for Global Health, University College, London, UK.

**Keywords:** COVID-19, Unemployment, Public Health, Containment, Lockdown, Fiscal Policy

## Abstract

**Background:** At the start of the coronavirus disease 2019 (COVID-19) pandemic, in the absence of pharmaceutical interventions, countries resorted to containment measures to stem the spread of the disease. In this paper, we have conducted a global study using a sample of 46 countries to evaluate whether these containment measures resulted in unemployment.

**Methods:** We use a difference-in-differences (DID) specification with a heterogenous intervention to show the varying intensity effect of containment measures on unemployment, on a sample of 46 countries. We explain variations in unemployment from January-June 2020 using stringency of containment measures, controlling for gross domestic product (GDP) growth, inflation rate, exports, cases of COVID-19 per million, COVID-19-specific fiscal spending, time fixed effects, region fixed effects, and region trends. We conduct further subset analyses by COVID-cases quintiles and gross national income (GNI) per capita quintiles.

**Results:** The median level of containment stringency in our sample was 43.7. Our model found that increasing stringency to this level would result in unemployment increasing by 1.87 percentage points (or 1.67 pp, after controlling for confounding). For countries with below median COVID-19 cases and below median GNI per capita, this effect is larger.

**Conclusion:** Containment measures have a strong impact on unemployment. This effect is larger in poorer countries and countries with low COVID-19 cases. Given that unemployment has profound effects on mortality and morbidity, this consequence of containment measures may compound the adverse health effects of the pandemic for the most vulnerable groups. It is necessary for governments to consider this in future pandemic management, and to attempt to alleviate the impact of containment measures via effective fiscal spending.

## Background

**Key Messages**

** Implications for policy makers**

- Using a multi-country sample, we found that a one-point increase in lockdown stringency was linked to 0.043 percentage point (pp) increase in unemployment, which could lead to a significant health consequence of its own.
- For low-income countries, the impact of containment measures was especially large, highlighting the disproportionate effect experienced by the poorer countries.
- The decision to impose stringent lockdown policy is paramount in saving lives, but it should be accompanied with policies that provide effective fiscal relief to alleviate the negative impact of the lockdown.

** Implications for the public**

Using a sample of 46 countries, this study analyses changes in unemployment in the early phase of the coronavirus disease 2019 (COVID-19) pandemic. While lockdowns can contain the spread of the virus, the adverse effect on the economy, in particular unemployment, has been raised as a key concern. The study result indicates that an increase in lockdown stringency is associated with an increase in unemployment. In this study, we discuss the importance of containing the spread of COVID-19 through stringent lockdown measures while also taking into consideration economic consequences to minimise death and unemployment until an effective vaccine/treatment could be developed.

In late 2019, a novel coronavirus (severe acute respiratory syndrome coronavirus 2, SARS-CoV-2) emerged in the Hubei province of China, causing an illness termed coronavirus disease 2019 (COVID-19).^1^ The rapid global spread of this virus has precipitated a multidimensional crisis. The World Health Organization (WHO) declared a pandemic in March 2020.^2^ As of January 2021, there have been over 85 million confirmed cases globally, with over 1.8 million deaths.^3^

 With no effective treatment or vaccine available, countries have resorted to a variety of public health measures in their attempt to contain the spread of the disease.^4,5^ These policies have sought to achieve social distancing through various restrictions such as closing workplaces and schools, banning public events, restricting gatherings, limiting internal movement within national boundaries, and closing international borders.

 Recent studies have highlighted the effectiveness of these interventions in limiting the spread of COVID-19 and thus, reducing mortality.^6-11^ Nonetheless, containment measures also result in economic and social costs in the form of negative externalities.^12^ As governments across the world enacted policies to slow or shut down economic activity, the global economy was expected to contract by almost 5%,^13^ which is a magnitude exceeding that of the 2008-2009 financial crisis.^14^

 This article will avoid presenting a dichotomy of the economy and public health. Rather, the emphasis will be on the importance of understanding the extent of the economic hardship, specifically unemployment, caused by the containment measures, which can have adverse health implications of its own.^15,16^ The aim of this research will be to evaluate whether containment measures result in unemployment. This article contributes to the literature evaluating the impacts of the containment measures, hoping to inform future pandemic management policies.^17^

 We will include an assessment of the variation in unemployment given different levels of containment, and how the impact varies given the national incidence of COVID-19 cases, fiscal expenditure, and national wealth. In the background section, we will discuss the existing evidence on the impact of COVID-19 on unemployment and the impact of unemployment on mortality. In the methods section, we will provide a detailed outline of the methods used to conduct the study analysis, followed by the results. In the discussion section, we will consider policy implications of the results, as well as study limitations. Finally, the conclusions will summarise the results and propose possible future research topics in this area.

 Several articles have evaluated the impact of COVID-19 and associated containment measures on unemployment and labour markets. Guerrieri et al^18^ considered the current economic crisis as a supply shock recession; however, they outlined how this could trigger a demand shortage that leads to reductions in output and employment greater than the original supply shock.

 Gupta et al^19^ and Bauer and Weber^20^ used the cross-state/region variation in the timing of lockdown implementation to measure the amount of unemployment caused by the lockdown in the U.S and Germany respectively. Employment rate fell by 7.2 percentage points (pp) in the United States and by 0.3 pp in Germany, as a result of the containment measures.

 Others have focused on the disparity in effect depending on job type and social economic status. Adams-Prassl et al^21^ noted that workers who cannot perform their work remotely were at greater risk of being unemployed or losing income. Both Mongey and Weinberg^22^ and Bick and Blandin^23^ found that people with lower levels of education and wealth were more likely to be in occupations that required significant face-to-face contact. As these occupations were more likely to be affected by social distancing measures, these workers faced the greatest threat of unemployment.

 Some studies have highlighted ambiguities as to whether unemployment can be solely attributed to containment measures. Kahn et al^24^ reported a large reduction in US job vacancies since March 2020. However, this effect was uniform across states despite their stay-at-home orders and spikes in the number of cases occurring at different times. Aum et al^25^ indicated that an increase in infection numbers could result in a similar rise in unemployment, even in the absence of a lockdown.

 This appears consistent with the hypothesis offered by Gourinchas^12^ that recession and subsequent unemployment are inevitable, even in the absence of containment measures. As the pandemic leads to economic uncertainty,^26^ this will cause precautionary responses from both firms and individuals, slowing the economy. A study by Goolsbee and Syverson^27^ showed how increased deaths from COVID-19 reduced consumer traffic, while containment measures simply shifted traffic from non-essential to essential retail.

 The extent of the loss in consumption could be significant. Hall et al^28^ estimate that society would be willing to forgo as much as 41% of its total consumption to avoid death from COVID-19, given an estimated mortality rate of 0.81%.^29^ Similarly, confronted with a decline in demand and uncertainty, firms could temporarily halt operations and/or reduce staff.^30,31^

 Our paper aims to contribute to the literature on the impacts of containment measure on unemployment. To our knowledge, this is the first multi-country study to do so. We focus on the impact when initial containment measures were implemented between March and June 2020, before pharmaceutical interventions were available.

## Methods

 Our analysis uses publicly available data from a sample of 46 countries. The outcome variable is the unemployment rate, while the explanatory variable is level of containment stringency. We use a difference-in-differences (DID) specification, with varying treatment intensity, to derive the effect of containment measures on unemployment. The following sections detail the data sources, sampling, model specification, and analyses conducted.

###  Data Sources

 For this study, we used publicly available secondary data collected from various sources. The dependent variable is the seasonally adjusted monthly unemployment rate by country, which is available from the Eurostat,^32^ the Organisation for Economic Co-operation and Development (OECD)databases,^33^ or each country’s official national statistics (See Supplementary file 1). The main independent variable is the stringency index of the government containment and closure policies. This index ranges from 0 (no measures) to 100 (full lockdown). This information is available from the Oxford COVID-19 Government Response Tracker (OxCGRT), which contains daily changes to the stringency index for 177 countries since January 1, 2020.^34^

 The OxCGRT also contains data on the monetary value of the fiscal stimuli employed by governments to lessen the economic impact of the pandemic. Additionally, in collaboration with OxCGRT, Our World in Data provides country level information including total population and total COVID-19 cases per million of population.^35^ Gross national income (GNI) per capita and exports as a percentage of gross domestic product (GDP) are available from the World Bank.^36^ Finally, real GDP growth (annual percentage change) and inflation rates can be found on the International Monetary Fund databank.^13^ Note that GNI, exports, GDP growth and inflation rates are from 2019. More details on the data sources can be found under Supplementary file 2.

###  Sampling Method

 We organised the study sample in panel data format, recording changes to the model variables on a bi-weekly basis. Out of the 177 countries contained in the OxCGRT dataset, a total of 46 countries (see Supplementary file 3) were selected to form the study sample. The inclusion criteria were defined based on the country’s unemployment data being available at least on a monthly basis. This excluded countries which release unemployment statistics on a quarterly or annual/semi-annual basis. The section below discusses how this variable was transformed to a bi-weekly basis. Additionally, we excluded countries with insufficient data for the covariates.

###  Model Specification

 We aim to evaluate the impact of containment measures, as measured by the OxCGRT containment measures stringency index, on unemployment. Because the containment measure variable is a continuous variable from 0 to 100, we used a model that can address the heterogeneity of intervention and capture its causal effect. We adapted a specification suggested by Banerjee et al^37^ where the intervention was a rate, expressed as a continuous variable ranging from 0 to 1. Therefore, the estimated coefficient for this variable shows the proportional effect of the intervention on the outcome variable. This is a commonly used DID design with a heterogenous intervention, which aims at capturing the effect of treatment with varying intensity.^38,39^ The final specification is as follows:


(1) $$Y_{ist}=\alpha +\delta_{s}+\gamma_{t}+\lambda_{st}+\beta Str_{ist}+Cov_{is}Ф+\varepsilon_{ist}$$

*Y*_ist_ is the outcome of interest, which is the rate of unemployment in each country *i*in each region *s*, and bi-weekly period *t*. *δ*_s_ are the region fixed effects, *γ*_t_ are bi-weekly period fixed effects, and *λ*_st_ are region trends. We include the region fixed effects and trends to control for region-wide unobserved time-varying effects, which allows us to strengthen the DID assumptions. *Cov*_is_ is a vector of time-fixed covariates, which include fiscal stimulus, COVID-19 cases per million, GDP growth, inflation rate, and exports as a percentage of GDP in 2019 for each country. The covariates are included to control for confounding. We will present the results with and without these covariates. *Str*_ist_ is the intervention, which is the level of containment stringency in each country at each bi-weekly period, as measured by the OxCGRT index. We have re-scaled the index (which ranges from 0 to 100) to a variable ranging from 0 to 1. The coefficient of interest is *β,* which provides the causal effect of containment stringency on unemployment. α is the constant and *ε*_ist_ is the error term. Each of the model parameters will be further explained in the next section.

###  Model Parameters

####  Outcome

 The outcome or dependent variable (*Y*_ist_ in equation 1) is the country-specific bi-weekly unemployment rate, recorded from January 2020 to June 2020. Unemployment data for the countries sampled were available monthly. Therefore, for each country, any two bi-weekly periods in the same month will have the same unemployment rate value. Because of this, we present the results with robust standard errors but also with clustered standard errors, at the country level, to account for autocorrelation.

####  Intervention

 The intervention is a continuous variable in the form of a standardised stringency index ranging from 0 (no intervention) to 100 (complete lockdown), which has fluctuated over the study period. This is comprised of nine different indicators on containment and closure policies, such as public information campaigns, school closures, workplace closures, and restrictions in movement, which can change daily (See Supplementary file 4 for the full list of indicators included). We re-scaled this to a variable ranging from 0 to 1 for the regression, and this appears as *str*_ist_ in equation 1.

 For the purposes of testing the parallel trends assumption needed in a DID design and the descriptive statistics, we defined treatment (high stringency) and control (low stringency) groups based on mean stringency over the study period. Countries with mean stringency exceeding the sample’s mean stringency (43.77 or 0.4377 in the rescaled stringency) were assigned to the high stringency group, while those below were assigned to the low stringency group. These groups would be equivalent to the “treatment” and “control” groups in a canonical DID design. In the specification with heterogenous treatment, however, we use a continuous treatment variable (ranging from 0 to 1), as discussed in the previous paragraph.

####  Time and Region Fixed Effects and Trends

 We included time fixed effects, using dummy variables for every bi-weekly period between January 2020 and June 2020. We used region fixed effects, with dummy variables based on the World Bank region classification. We then include region trends, by interacting the time and region fixed effects variables. This helps account for any unobserved region-specific time-varying effects, such as joint regional policies (ie, European Union common pandemic policies), to strengthen the DID assumptions.

####  Additional Covariates

 As shown in equation 1, we included a matrix of relevant time fixed covariates denoted by *Cov*_is_, which will be discussed as follows:

*GDP growth in 2019:* It is widely accepted that GDP growth is one of the most important macroeconomic predictors for forecasting unemployment.^40,41^ However, the effect is not always instant, as labour market flexibility can delay changes being reflected in the unemployment rate^42^. This potential delay makes it particularly important to control for 2019 GDP growth. The expected effect on unemployment is *negative*, ie, higher GDP growth will reduce unemployment.

*Inflation rate in 2019:* The relationship between inflation and unemployment has been well documented, where increased inflation is associated with lower unemployment.^43^ However, in the presence of exogenous shocks (such as a global pandemic), stagflation can occur whereby inflation and unemployment rise jointly. As with GDP, a possible delay in the effect means that the inflation rate in 2019 could have an effect on unemployment in 2020. The expected effect on unemployment is *ambiguous*.

*Value of exports as a percentage of GDP in 2019:* Export of goods and services as a percentage of GDP from 2019 was included in the model to determine the level of dependency on foreign markets. Higher exports are associated with higher GDP, which could lead to a reduction in unemployment.^44^ However, depending on labour flexibility, increased exports may result in higher short-term frictional unemployment as the labour market readjusts to the expanding sector.^45^ Further, during a global pandemic, a country that is highly dependent on foreign exports may be more likely to experience an economic downturn, leading to unemployment. For this reason, the expected effect of exports on unemployment is ambiguous.

*COVID-19 Fiscal stimulus as a percentage of GDP (total between January-June 2020): * In response to the COVID-19 health crises, governments have implemented fiscal packages to lessen the impact of the economic downturn.^46^ Notwithstanding the details of each country’s policies, it is expected that the larger the fiscal spending, the greater the reduction in unemployment.^47^ Initial modelling has shown that fiscal spending can stem the rise in unemployment, albeit moderately.^48^ Therefore, the impact of fiscal stimulus on unemployment is expected to be *negative*.

*COVID-19 cases per million population (total between January-June 2020):* Although all countries in the sample have been exposed to the virus, the extent of infection differs greatly from country to country. High incidence of COVID-19 is expected to lead to higher unemployment, due to precautionary responses from firms reducing operations and individuals fearing contracting the novel virus reducing consumer traffic.^12,27^ The impact of the cumulative incidence of the virus on unemployment is predicted to be *positive*.

###  Statistical and Regression Analysis

####  Descriptive Statistics

 We present the mean and standard deviation for all the model variables, divided between low stringency and high stringency countries. There are 21 high stringency countries and 25 low stringency countries. We present summary statistics for the outcome and treatment variables, before and after March 16, 2020. This date is the closest biweekly period to the date when the WHO officially declared a pandemic (March 11, 2020).^2^

####  Parallel Trends Assessment

 Given that this is a DID design, we test the parallel trends assumption graphically. As discussed before, the high stringency and low stringency groups correspond to treatment and control groups. Similarly, we use March 16, 2020 as discussed in the previous section, to determine the pre- and post-treatment periods.

####  Main Regression

 The main specification is outlined in equation 1, where *β* is the coefficient of interest giving the impact of containment measures on unemployment. Since the containment stringency index has been rescaled in the specification, the coefficient will also need to be rescaled for interpretation. To do this, we divided the coefficient by 100 to find the effect of 1 point of stringency on unemployment percentage points.

####  Subset Analysis

 Finally, we conducted four subset analyses, to determine the impact of containment measures on unemployment, conditional on COVID-19 cases and national income. We applied the model using equation 1 for two separate subsets: countries with above median and below median number of cases of COVID-19. The same procedure was followed on two subsets of above median and below median national income, as measured by GNI per capita.

## Results

###  Descriptive Statistics

Table 1summarises the basic descriptive statistics of the model variables for both the low and high stringency countries. For the time-varying variables (unemployment rate and stringency) we use data for 46 countries over 12 two-week periods (24 weeks). This yields 552 observations, out of which 252 relate to 21 high stringency countries, and 300 relate to 25 low stringency countries. As described before, we only use this separation of high and low stringency countries for the descriptive statistics and parallel trends assessment. In the regression, we do not use this distinction and rather implement a continuous treatment variable.

**Table 1:** Descriptive Statistics Comparison of Treatment (High Stringency) and Control (Low Stringency) Countries

**Variables**	**High Stringency**		**Low Stringency**	
	**Mean**	**SD**	**Mean**	**SD**
Unemployment rate (pre-pandemic)	6.69	0.18	5.42	0.14
Unemployment rate (post-pandemic)	8.65	0.42	6.73	0.24
Containment stringency (pre-pandemic)	0.13	0.02	0.06	0.01
Containment stringency (post-pandemic)	0.75	0.01	0.63	0.01
GDP growth	2.64	0.35	1.95	0.24
Inflation rate	2.50	0.71	2.25	0.29
Exports (% of GDP)	48.13	8.63	60.37	7.47
Fiscal stimulus (% of GDP)	0.07	0.01	0.06	0.01
COVID-19 cases (per million)	3352.31	600.16	2594.46	624.42

Abbreviations: COVID-19, coronavirus disease 2019; SD, standard deviation; GDP, gross domestic product.

 Unemployment rate was higher for high stringency countries both in the pre- and post-pandemic periods (defined as March 16, 2020). However, while the unemployment rate increased by just over 1 pp for low stringency countries, this increase was almost 2 pp for high stringency countries. As expected, the stringency level is higher in high stringency countries pre- and post-pandemic. GDP growth, and COVID-19 cases were higher in the high stringency group compared to the low stringency group. Exports were higher in the low stringency group, compared to the high stringency group. Inflation rate and fiscal stimulus were comparable across both groups.

###  Regression Analysis

 We tested the parallel trends assumption graphically, shown in Figure. The vertical red line denotes the date (March 16, 2020) we used to separate the pre- and post-treatment periods, which is the closest to the pandemic declaration date. The treatment (high stringency) and control (low stringency) groups had parallel trends prior to this date. In the post-treatment period, both groups experienced an increase in unemployment, but the high stringency’s group increase was greater. As discussed before, although we use a binary treatment in testing the parallel trends assumption, we allow for a continuous treatment (from 0 to 1) in the regression.

**Figure:**
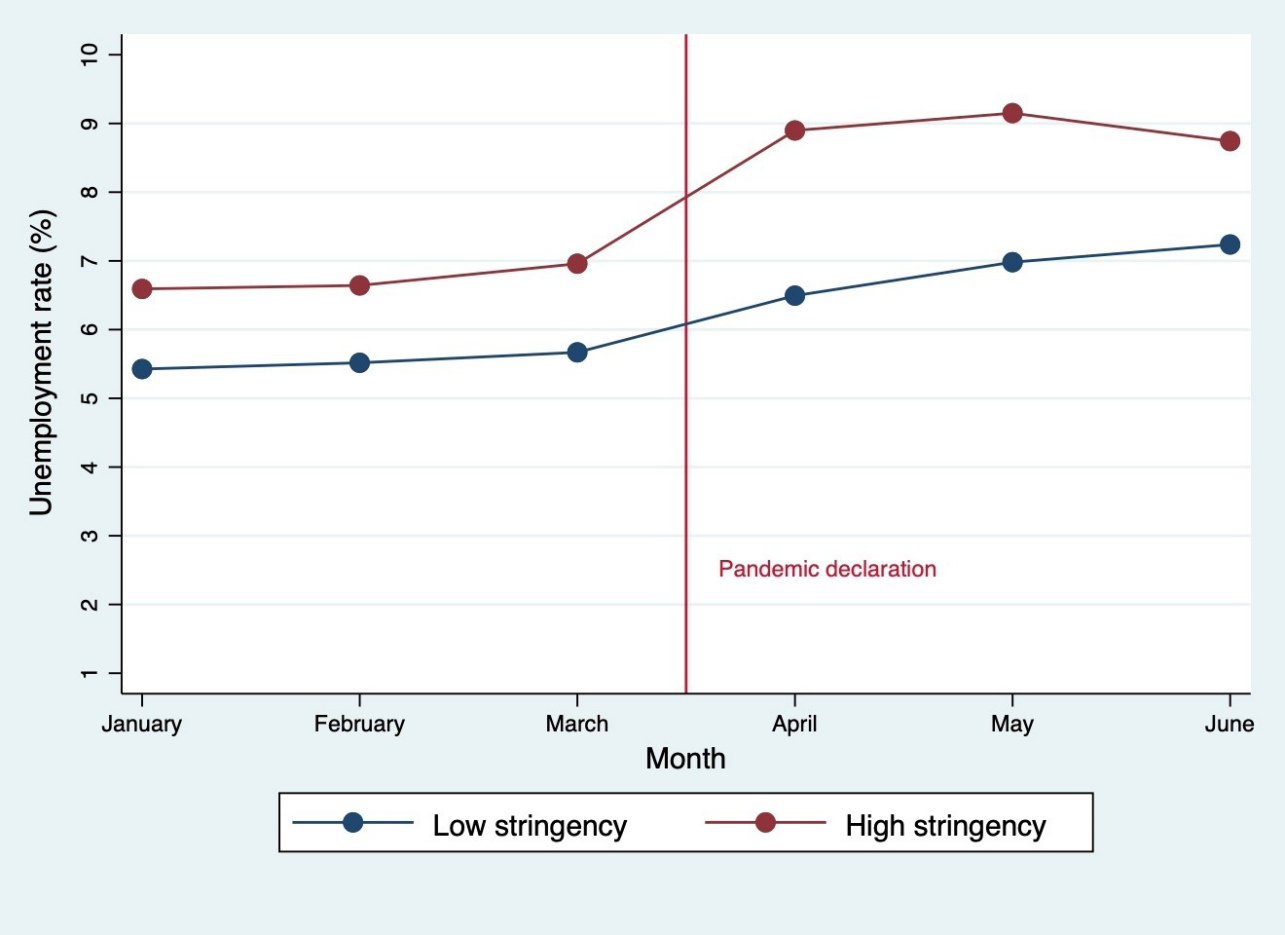
Parallel Trends.

Table 2summarises the results from the model defined under equation 1. We present four different estimations: (1) and (3) use robust standard errors, while (2) and (4) use clustered standard errors. We include covariates (GDP growth, inflation rate, exports, fiscal stimulus, and COVID-19 cases) in estimations (3) and (4).

**Table 2:** Regression Results

**Variable**	**(1)**	**(2)**	**(3)**	**(4)**
	**Without covariates** **Robust SE**	**Without Covariates** **Clustered SE**	**With Covariates ** **Robust SE**	**With Covariates ** **Clustered SE**
Containment stringency	4.29*** (1.05)	4.29** (1.94)	3.83*** (1.03)	3.83** (1.82)
GDP growth	N/A	N/A	0.01 (0.09)	0.01 (0.31)
Inflation rate	N/A	N/A	0.17** (0.08)	0.17 (0.28)
Fiscal spending (% of GDP)	N/A	N/A	-6.40** (2.80)	-6.40 (9.45)
COVID-19 cases per million	N/A	N/A	0.0001* (0.00006)	0.0001 (0.002)
Exports (% of GDP)	N/A	N/A	-0.02*** (0.004)	-0.02 (0.02)
R^2^	0.40	0.40	0.46	0.46
N	552	552	552	552

Abbreviations: COVID-19, coronavirus disease 2019; SE, standard error; GDP, gross domestic product. Standard errors in parentheses. * *P* <.1, ** *P* <.05, *** *P* <.01. We have also controlled for bi-weekly period fixed effects, region fixed effects, and region trends with coefficients not shown in this table for clarity.

 Under all estimations, containment stringency increases the unemployment rate. This result is robust to adding covariates and clustering standard errors, and it is significant at the 5% level. An increase of one point of containment stringency increases unemployment by between 0.043 and 0.038 pp. The robustness checks using a balanced time period and development status group fixed effects showed that these results remain consistent. In other words, keeping all else constant, an increase in stringency from 0 to 43.77 (the sample’s mean stringency), as measured by the OxCGRT index, would, on average, increase unemployment by 1.67 to 1.87 pp. Although there are no known studies that have analysed the global effect of the containment and closure measures on unemployment, the findings are consistent with Bauer and Weber^20^ and Gupta et al^19^ who found that these measures increased unemployment rates in Germany by 0.3 pp and the United States by 7.2 pp, respectively. Our figures, being a multi-country average, lie in between these estimates.

 Other statistically significant covariates, at the 5% significance level, included inflation rate, fiscal spending as a percentage of GDP, COVID-19 cases per million, and exports as a percentage of GDP. However, this significance disappears for all these variables after clustering standard errors. Exports and fiscal spending are associated with a reduction in unemployment, while inflation rate and COVID-19 cases are associated with an increase in unemployment.

Table 3 shows the results of the subset analyses only displaying the coefficient for the treatment variable for clarity.

 Containment stringency continues to have a significant impact on unemployment in the subset analysis. For countries with below median COVID-19 cases, the coefficient is larger than for countries with above median COVID-19 cases. In countries where increased COVID-19 cases have not caused wide economic uncertainty, containment measures might be counterproductive for the economy.

**Table 3:** Subset Regression Results

**Variable**	**(1)**	**(2)**	**(3)**	**(4)**
	**Without covariates** **Robust SE**	**Without covariates** **Clustered SE**	** With covariates** **Robust SE**	** With covariates** **Clustered SE**
**COVID-19 Cases Subset**				
Below median cases – Containment stringency	5.529*** (1.63)	5.529 (3.39)	1.477 (1.46)	1.477 (1.75)
Above median cases – Containment stringency	3.583** (1.58)	3.583 (3.08)	3.5** (1.61)	3.5 (3.10)
**GNI Per Capita Subset**				
Below median income – Containment stringency	5.931*** (1.93)	5.931* (3.28)	3.017 (2.03)	3.017 (3.32)
Above median income – Containment stringency	1.984 (1.46)	1.984 (3.25)	0.309 (1.19)	0.309 (2.37)

Abbreviations: COVID-19, coronavirus disease 2019; SE, standard error; GNI, gross national income. Standard errors in parentheses. * *P* <.1, ** *P* <.05, *** *P* <.01. We have also controlled for bi-weekly period fixed effects, region fixed effects, region trends, and other covariates (omitting COVID-19 cases in the COVID-19 subset) with coefficients not shown in this table for clarity.

 Further, for countries with below median GNI per capita, the coefficient is larger than in the main results and significant. These countries may be more vulnerable to workplace closures and may not have the infrastructure for remote working. These results must be taken with caution as they are not robust to adding covariates or clustering standard errors.

## Discussion

###  Policy Implications

 We have shown that stronger containment measures are associated with increased unemployment, using a multi-country sample. This has repercussions that go beyond the economy and can result in poor health outcomes. The importance of reducing unemployment, as part of an integrated approach to better public health, cannot be overstated. There is significant literature on the adverse physical and mental health effects associated with unemployment, which can lead to higher mortality risk.^49-51^ A study by Montgomery et al^52^ found that the hazard ratio for all-cause mortality can be up to 3.44 times greater for the unemployed individual, compared to the employed individual, with age and length of unemployment as important effect amplifiers.

 Voss et al^53^ analysed all-cause mortality for 1067 pairs of twins, where one of the twins had experienced unemployment while the other had not. The sample of twins aimed to control for biological confounders. The study estimated the relative risk of death to be 1.5 times higher for the unemployed twin. These findings illustrate the serious health consequences of unemployment, and how health policies need to consider this outcome.

 Further, a recent study modelling the impact of macroeconomic conditions on chronic health in the United Kingdom, showed that the latter are poorer during periods of high unemployment.^54^ Since many chronic health conditions increase the risk of COVID-19 severe disease,^55^ this could present a dangerous feedback loop if the disease ultimately becomes endemic.^56^

 Additionally, while our study evaluated the overall impact at the national level, the effect is likely to be felt unevenly across the population. Epidemiological studies have shown that the pandemic has had disproportionate health impacts on ethnic minorities and people on low incomes.^57-61^ Existing literature indicates that periods of economic recession result in poorer health outcomes for already disadvantaged groups.^62^ Finally, studies in the United States and United Kingdom show that the unemployment shock from the pandemic has been strongest for those in ethnic minorities and low-income groups.^63,64^ This multi-dimensional impact for vulnerable groups of increased disease severity from COVID-19, greater risk of unemployment from containment measures, and poorer health outcomes from an economic recession could further existing inequalities.^65^

 At the country level, our study showed that the effect on unemployment was especially large for countries with the lowest GNI per capita. Given the fragile state of many of the poorest economies, there are plausible reasons for thinking that they would suffer most severely,^66^ highlighting their need for support from the international community. We propose that public policy needs to consider the many dimensions and determinants of health. Containment measures are necessary and effective tools in limiting the spread of the virus. However, when using these measures, other policies must be implemented to alleviate their negative impact, especially on the most vulnerable individuals or countries.^67^

###  Limitations of This Study

 The study is subject to data limitations. For example, testing and data collection capabilities differ greatly from country to country which may affect the number of COVID-19 cases recorded. Further, many of the newly unemployed are not actively looking for work due to current circumstances,^68^ indicating that the unemployment data may not be an accurate measure of employment losses. Only 46 countries were included in the study sample due to limited availability of short-term unemployment data. Furthermore, the post-treatment period was relatively short at 3.5 months. After July 2020, initial containment measures were lifted, which was followed by several new pandemic waves, and eventually pharmaceutical interventions. We wanted to show the impact of the initial shock of containment measures, before further complexities arose.

 The size and features of the sample also limit the generalisability of this study. There is an overrepresentation of developed economies in the sample data. This is particularly relevant for the results of the subset analysis using countries with lowest quintile of GNI per capita, which are not comparable to the lowest GNI per capita quintile in the population. For instance, the country with the lowest GNI per capita in the sample was India (US$ 6960), which has a GNI per capita 792% higher than the poorest country in the population, Burundi (US$ 780).^36^ Generalisation of the results to the poorest countries must be made with caution due to the fundamental differences implied by the income levels.

 The analysis treats every point in the stringency index as equal. As outlined in Supplementary file 4, the intervention stringency is derived from nine evenly weighted categorical indicators. However, some indicators are likely to impact unemployment more than others; for example, workplace closure is likely to cause more unemployment than a public health campaign.^69^

 Therefore, the effect on unemployment from 10 stringency points consisting solely of a public health campaign is likely to differ greatly from 10 stringency points caused by workplace closures. While this is an important limitation, it is also reasonable to assume logical ordering of the indicators of the stringency index with measures believed to be socially and economically more harmful introduced later. However, the extent to which this could alleviate the limitation depends on the similarity of this ordering from country to country.

 Finally, our research does not provide any indication on whether the length of time that the containment measures are in place matter, or for how long is the impact sustained.

## Conclusion

 We investigated the impact of containment measures on unemployment during the COVID-19 pandemic. We showed that there is a positive and significant impact of containment on unemployment, using a multi-country sample. The impacts are most strongly felt by low-income countries, and the literature shows that there are also disparities within countries, with the largest impact for the most vulnerable individuals. Given the results of the subset analyses, we also propose that the optimal policy is to prioritise containment measures in a situation of high COVID-19 prevalence, but to carefully consider the impacts of these measures in a situation of low COVID-19 prevalence.

 These results are important in framing the policy response for future pandemics. Given the inevitability of future pandemics,^70^ it is essential that policymakers ensure preparedness while pharmaceutical interventions, such as vaccines or antiviral drugs, remain unavailable. Containment measures have been shown as effective measures in these initial stages of the pandemic, but they come with costs. To ensure the health and welfare of individuals, these measures need to be applied in a considered manner, taking into account the specific local circumstances. This article aimed to give an overview of what these considerations may be and provide a starting point for further analyses.

 The results presented in the subset analyses present an interesting starting point for future research. The effects of containment measures do not seem to be uniform across countries, and indeed may be affected by a variety of national characteristics. Future research should explore, with a larger sample, whether these results are robust. Specifically, the question of how COVID-19-specific fiscal spending can impact unemployment, for varying levels of COVID-19 cases and GNI per capita, should be further researched.

 As mentioned in the limitations, we considered the complete stringency index and did not investigate specific stringency indicators. Further research could evaluate whether specific containment measures have a stronger impact on unemployment than others. This should be helpful in guiding policy, as it could help determine when to most effectively apply fiscal policy to stem rising unemployment, depending on the containment measures implemented.

 Finally, more research is needed on the duration of this effect. Most of the literature around the health risks of unemployment focuses on medium- to long-term unemployment. If containment measures result in short-term unemployment, the concerning health consequences might not bear out. However, if the effect on unemployment is sustained, this will be an important result to consider for the management of future pandemics.

## Ethical issues

 The research did not require ethical approval, as it pertained analysis of publically available secondary country-level data.

## Competing interests

 Authors declare that they have no competing interests.

## Authors’ contributions

 AC and WM conceived and designed the study. WM acquired the data. AC, WM, and RL analysed the data and drafted the manuscript.

## Supplementary files


Supplementary file 1. Unemployment Data Sources by Country.

Supplementary file 2. Data Source for Individual Covariates.

Supplementary file 3. List of Sample Countries by Treatment Groups (N = 46).

Supplementary file 4. Additional Detail on Stringency Index.
