# Supplementary material for: Impact of COVID-19 Containment Measures on Unemployment: A Multi-country Analysis Using a Difference-in-Differences Framework
Source: Int J Health Policy Manag. 2023 Jan 31;12:7036. doi: 10.34172/ijhpm.2022.7036 (PMC10125098; doi:10.34172/ijhpm.2022.7036)
Supplement: Supplementary file 1 — Unemployment Data Sources by Country. [file ijhpm-12-7036-s001.pdf]

Article title: Impact of COVID-19 Containment Measures on Unemployment: A Multi-country Analysis Using a Difference-in-Differences Framework

Journal name: International Journal of Health Policy and Management (IJHPM)

Authors' information: Walter Morris, Ana Correa*, Rolando Leiva

Institute for Global Health, University College, London, UK.

(*Corresponding author: a.correa@ucl.ac.uk)

Supplementary file 1. Unemployment Data Sources by Country

Countries	Data Source	Link
Australia	OECD	https://data.oecd.org/unemp/unemployment-rate.htm
Austria	OECD	https://data.oecd.org/unemp/unemployment-rate.htm
Belgium	OECD	https://data.oecd.org/unemp/unemployment-rate.htm
Brazil	IBGE	https://www.cut.org.br/indicadores/desemprego-mensal
Bulgaria	Eurostat	https://ec.europa.eu/eurostat/web/lfs/data/database
Canada	OECD	https://data.oecd.org/unemp/unemployment-rate.htm
Chile	OECD	https://data.oecd.org/unemp/unemployment-rate.htm
China	NBS	https://data.stats.gov.cn/english/easyquery.htm?cn=A01
Colombia	OECD	https://data.oecd.org/unemp/unemployment-rate.htm
Croatia	Eurostat	https://ec.europa.eu/eurostat/web/lfs/data/database
Cyprus	Eurostat	https://ec.europa.eu/eurostat/web/lfs/data/database
Czech Republic	OECD	https://data.oecd.org/unemp/unemployment-rate.htm
Denmark	OECD	https://data.oecd.org/unemp/unemployment-rate.htm
Estonia	OECD	https://data.oecd.org/unemp/unemployment-rate.htm
Finland	OECD	https://data.oecd.org/unemp/unemployment-rate.htm
France	OECD	https://data.oecd.org/unemp/unemployment-rate.htm
Germany	OECD	https://data.oecd.org/unemp/unemployment-rate.htm
Greece	OECD	https://data.oecd.org/unemp/unemployment-rate.htm
Hong Kong	CSD	https://www.censtatd.gov.hk/hkstat/sub/sp200.jsp?ID=0&productType=8&tableID=006
Hungary	OECD	https://data.oecd.org/unemp/unemployment-rate.htm
Iceland	OECD	https://data.oecd.org/unemp/unemployment-rate.htm
India	CMIE	https://unemploymentinindia.cmie.com/kommon/bin/sr.php?kall=wshowtab&tabno=0001
Ireland	OECD	https://data.oecd.org/unemp/unemployment-rate.htm

Israel	OECD	https://data.oecd.org/unemp/unemployment-rate.htm
Italy	OECD	https://data.oecd.org/unemp/unemployment-rate.htm
Japan	OECD	https://data.oecd.org/unemp/unemployment-rate.htm
Korea	OECD	https://data.oecd.org/unemp/unemployment-rate.htm
Latvia	OECD	https://data.oecd.org/unemp/unemployment-rate.htm
Lithuania	OECD	https://data.oecd.org/unemp/unemployment-rate.htm
Luxembourg	OECD	https://data.oecd.org/unemp/unemployment-rate.htm
Malaysia	DSM	https://www.dosm.gov.my/v1/index.php?r=column/cthemeByCat&cat=124&bul_id=Z0M1dy9sQjYrMXBTNVpEVE5HVWt5Zz09&menu_id=Tm8zcnRjdVRNWWlpWjRlbmtlaDk1UT09
Mexico	OECD	https://data.oecd.org/unemp/unemployment-rate.htm
Netherlands	OECD	https://data.oecd.org/unemp/unemployment-rate.htm
Norway	OECD	https://data.oecd.org/unemp/unemployment-rate.htm
Peru	BCRP	https://estadisticas.bcrp.gob.pe/estadisticas/series/mensuales/resultados/PN38063GM/html?fbclid=IwAR0FP5RKACoxMRC3Kyxca5a1W0whd8LvC29tQ3yD2l_jHKnCWa41ZNWqQAA
Philippines	PSA	https://psa.gov.ph/content/employment-situation-october-2019
Poland	OECD	https://data.oecd.org/unemp/unemployment-rate.htm
Portugal	OECD	https://data.oecd.org/unemp/unemployment-rate.htm
Romania	Eurostat	https://ec.europa.eu/eurostat/web/lfs/data/database
Russia	FSS	https://eng.gks.ru/ (direct link)
Singapore	MMS	https://stats.mom.gov.sg/Pages/UnemploymentTimeSeries.aspx
Slovak Republic	OECD	https://data.oecd.org/unemp/unemployment-rate.htm
Slovenia	OECD	https://data.oecd.org/unemp/unemployment-rate.htm
Spain	OECD	https://data.oecd.org/unemp/unemployment-rate.htm
Sweden	OECD	https://data.oecd.org/unemp/unemployment-rate.htm
Switzerland	Eurostat	https://ec.europa.eu/eurostat/web/lfs/data/database
Taiwan	NSRC	https://eng.stat.gov.tw/ct.asp?xItem=42761&ctNode=1609&mp=5
Turkey	OECD	https://data.oecd.org/unemp/unemployment-rate.htm
United Kingdom	OECD	https://data.oecd.org/unemp/unemployment-rate.htm
United States	OECD	https://data.oecd.org/unemp/unemployment-rate.htm
Uruguay	INEU	https://ine.gub.uy/indicadores?indicadorCategoryId=67534&fbclid=IwAR0EBFhq_U8QXqjjMEioKJmmQLP6L2Viiuv7JsIWITECZzPWHTIfPkigb1k
