# Supplementary material for: Impact of COVID-19 Containment Measures on Unemployment: A Multi-country Analysis Using a Difference-in-Differences Framework
Source: Int J Health Policy Manag. 2023 Jan 31;12:7036. doi: 10.34172/ijhpm.2022.7036 (PMC10125098; doi:10.34172/ijhpm.2022.7036)
Supplement: Supplementary file 2 — Data Source for Individual Covariates. [file ijhpm-12-7036-s002.pdf]

Article title: Impact of COVID-19 Containment Measures on Unemployment: A Multi-country Analysis Using a Difference-in-Differences Framework

Journal name: International Journal of Health Policy and Management (IJHPM)

Authors' information: Walter Morris, Ana Correa*, Rolando Leiva

Institute for Global Health, University College, London, UK.

(*Corresponding author: a.correa@ucl.ac.uk)

Supplementary file 2. Data Source for Individual Covariates

**Variables**	**Data Source**	**Access Date**	**Web link**
Government Stringency Index	OxCGRT	14 Jul 2020	https://www.bsg.ox.ac.uk/research/research-projects/coronavirus-government-response-tracker
Total Fiscal Stimulus	OxCGRT	14 Jul 2020	https://www.bsg.ox.ac.uk/research/research-projects/coronavirus-government-response-tracker
2019 GDP growth	IMF	27 Nov 2020	https://www.imf.org/external/datamapper/datamapper/PCPIPCH@WEO/OEMDC/ADVEC/WEOWORLD
2019 Inflation rate	IMF	27 Nov 2020	https://www.imf.org/external/datamapper/NGDP_RPCH@WEO/OEMDC/ADVEC/WEOWORLD
2019 Export (% of GDP)	World Bank	27 Nov 2020	https://data.worldbank.org/indicator/NE.EXP.GNFS.ZS
COVID-19 Cases per One Million Population	OWID	10 Aug 2020	https://github.com/owid/covid-19-data/tree/master/public/data
GDP, PPP (current international $)	World Bank	28 Nov 2020	https://data.worldbank.org/indicator/NY.GDP.MKTP.PP.CD
GDP, PPP (current international $) Taiwan*	IMF	20 Dec 2020	https://www.imf.org/external/datamapper/PPPGDP@WEO/TWN
Country Regions	World Bank via OWID	29 Nov 2020	https://ourworldindata.org/world-region-map-definitions
Country Development Status	UN	27 Nov 2020	https://unctadstat.unctad.org/en/classifications.html

*World Bank data does not account Taiwan therefore IMF data was used.
