# Supplementary material for: Impact of COVID-19 Containment Measures on Unemployment: A Multi-country Analysis Using a Difference-in-Differences Framework
Source: Int J Health Policy Manag. 2023 Jan 31;12:7036. doi: 10.34172/ijhpm.2022.7036 (PMC10125098; doi:10.34172/ijhpm.2022.7036)
Supplement: Supplementary file 3 — List of Sample Countries by Treatment Groups (N = 46). [file ijhpm-12-7036-s003.pdf]

Article title: Impact of COVID-19 Containment Measures on Unemployment: A Multi-country Analysis Using a Difference-in-Differences Framework

Journal name: International Journal of Health Policy and Management (IJHPM)

Authors' information: Walter Morris, Ana Correa*, Rolando Leiva

Institute for Global Health, University College, London, UK.

(*Corresponding author: a.correa@ucl.ac.uk)

Supplementary file 3. List of Sample Countries by Treatment Groups (N = 46)

High stringency Group (N=21)	Low stringency Group (N=25)
Belgium	Australia
Brazil	Austria
China	Bulgaria
Colombia	Canada
Croatia	Chile
Cyprus	Czech Republic
France	Denmark
India	Estonia
Ireland	Finland
Italy	Germany
Korea	Greece
Malaysia	Hungary
Peru	Iceland
Poland	Latvia
Portugal	Lithuania
Romania	Luxembourg
Russia	Mexico
Singapore	Netherlands
Spain	Norway
Turkey	Slovak Republic
United States	Slovenia
	Sweden
	Switzerland
	United Kingdom
	Uruguay
