# Supplementary material for: Impact of COVID-19 Containment Measures on Unemployment: A Multi-country Analysis Using a Difference-in-Differences Framework
Source: Int J Health Policy Manag. 2023 Jan 31;12:7036. doi: 10.34172/ijhpm.2022.7036 (PMC10125098; doi:10.34172/ijhpm.2022.7036)
Supplement: Supplementary file 4 — Additional Detail on Stringency Index. [file ijhpm-12-7036-s004.pdf]

Article title: Impact of COVID-19 Containment Measures on Unemployment: A Multi-country Analysis Using a Difference-in-Differences Framework

Journal name: International Journal of Health Policy and Management (IJHPM)

Authors' information: Walter Morris, Ana Correa*, Rolando Leiva

Institute for Global Health, University College, London, UK.

(*Corresponding author: a.correa@ucl.ac.uk)

Supplementary file 4. Additional Detail on Stringency Index

The stringency index is comprised of nine individual component indicators summarised in below table. The different ordinal scales in the indicators are normalised to produce a sub-index score ranging from 0 (no measures) to 100 (full lockdown). Further information on the stringency index is available from GitHub using the following links.

Information on individual indicators:

https://github.com/OxCGRT/covid-policy-tracker/blob/master/documentation/codebook.md

Information on the index calculation:

https://github.com/OxCGRT/covid-policy-tracker/blob/master/documentation/index_methodology.md

Below table (adapted from OxCGRT[34]) summarises the nine component indicators of the stringency index:

ID	Name	Description	Measurement	Coding
C1	C1_School closing	Record closings of schools and universities	Ordinal scale	0 - no measures
				1 - recommend closing or all schools open with alterations resulting in significant differences compared to non-Covid-19 operations
				2 - require closing (only some levels or categories, eg just high school, or just public schools)
				3 - require closing all levels
				Blank - no data
	C1_Flag		Binary flag for geographic scope	0 - targeted
				1- general

				Blank - no data
C2	C2_Workplace closing	Record closings of workplaces	Ordinal scale	0 - no measures
				1 - recommend closing (or recommend work from home) or all businesses open with alterations resulting in significant differences compared to non-Covid-19 operation
				2 - require closing (or work from home) for some sectors or categories of workers
				3 - require closing (or work from home) for all-but-essential workplaces (eg grocery stores, doctors)
				Blank - no data
	C2_Flag		Binary flag for geographic scope	0 - targeted
				1- general
				Blank - no data
C3	C3_Cancel public events	Record cancelling public events	Ordinal scale	0 - no measures
				1 - recommend cancelling
				2 - require cancelling
				Blank - no data
	C3_Flag		Binary flag for geographic scope	0 - targeted
				1- general
				Blank - no data
C4	C4_Restrictions on gatherings	Record limits on gatherings	Ordinal scale	0 - no restrictions
				1 - restrictions on very large gatherings (the limit is above 1000 people)
				2 - restrictions on gatherings between 101-1000 people
				3 - restrictions on gatherings between 11-100 people
				4 - restrictions on gatherings of 10 people or less
				Blank - no data
	C4_Flag		Binary flag for geographic scope	0 - targeted
				1- general
				Blank - no data
C5	C5_Close public transport	Record closing of public transport	Ordinal scale	0 - no measures
				1 - recommend closing (or significantly reduce volume/route/means of transport available)
				2 - require closing (or prohibit most citizens from using it)
				Blank - no data

	C5_Flag		Binary flag for geographic scope	0 - targeted<br>1- general<br>Blank - no data
C6	C6_Stay at home requirements	Record orders to "shelter-in-place" and otherwise confine to the home	Ordinal scale	0 - no measures<br>1 - recommend not leaving house<br>2 - require not leaving house with exceptions for daily exercise, grocery shopping, and 'essential' trips<br>3 - require not leaving house with minimal exceptions (eg allowed to leave once a week, or only one person can leave at a time, etc)<br>Blank - no data
	C6_Flag		Binary flag for geographic scope	0 - targeted<br>1- general<br>Blank - no data
C7	C7_Restrictions on internal movement	Record restrictions on internal movement between cities/regions	Ordinal scale	0 - no measures<br>1 - recommend not to travel between regions/cities<br>2 - internal movement restrictions in place<br>Blank - no data
	C7_Flag		Binary flag for geographic scope	0 - targeted<br>1- general<br>Blank - no data
C8	C8_International travel controls	Record restrictions on international travel<br><br>Note: this records policy for foreign travellers, not citizens	Ordinal scale	0 - no restrictions<br>1 - screening arrivals<br>2 - quarantine arrivals from some or all regions<br>3 - ban arrivals from some regions<br>4 - ban on all regions or total border closure<br>Blank - no data
H1	H1_Public information campaigns	Record presence of public info campaigns	Ordinal scale	0 - no Covid-19 public information campaign<br>1 - public officials urging caution about Covid-19<br>2- coordinated public information campaign (eg across traditional and social media)<br>Blank - no data
